# Guanylyl cyclase/natriuretic peptide receptor-A signaling antagonizes phosphoinositide hydrolysis, Ca^2+^ release, and activation of protein kinase C

**DOI:** 10.3389/fnmol.2014.00075

**Published:** 2014-08-22

**Authors:** Kailash N. Pandey

**Affiliations:** Department of Physiology, School of Medicine, Tulane University Health Sciences CenterNew Orleans, LA, USA

**Keywords:** natriuretic peptides, natriuretic peptide receptors, membrane guanylyl cyclases, cGMP, cAMP, Ca^2+^, inositol triphosphate

## Abstract

Thus far, three related natriuretic peptides (NPs) and three distinct sub-types of cognate NP receptors have been identified and characterized based on the specific ligand binding affinities, guanylyl cyclase activity, and generation of intracellular cGMP. Atrial and brain natriuretic peptides (ANP and BNP) specifically bind and activate guanylyl cyclase/natriuretic peptide receptor-A (GC-A/NPRA), and C-type natriuretic peptide (CNP) shows specificity to activate guanylyl cyclase/natriuretic peptide receptor-B (GC-B/NPRB). All three NPs bind to natriuretic peptide receptor-C (NPRC), which is also known as clearance or silent receptor. The NPRA is considered the principal biologically active receptor of NP family; however, the molecular signaling mechanisms of NP receptors are not well understood. The activation of NPRA and NPRB produces the intracellular second messenger cGMP, which serves as the major signaling molecule of all three NPs. The activation of NPRB in response to CNP also produces the intracellular cGMP; however, at lower magnitude than that of NPRA, which is activated by ANP and BNP. In addition to enhanced accumulation of intracellular cGMP in response to all three NPs, the levels of cAMP, Ca^2+^ and inositol triphosphate (IP_3_) have also been reported to be altered in different cells and tissue types. Interestingly, ANP has been found to lower the concentrations of cAMP, Ca^2+^, and IP_3_; however, NPRC has been proposed to increase the levels of these metabolic signaling molecules. The mechanistic studies of decreased and/or increased levels of cAMP, Ca^2+^, and IP_3_ in response to NPs and their receptors have not yet been clearly established. This review focuses on the signaling mechanisms of ANP/NPRA and their biological effects involving an increased level of intracellular accumulation of cGMP and a decreased level of cAMP, Ca^2+^, and IP_3_ in different cells and tissue systems.

## INTRODUCTION

Atrial natriuretic factor/peptide (ANF/ANP) is produced and secreted in the specific granules of cardiac atrial myocytes, which participates in the control of extracellular fluid volume, electrolyte balance, and mean arterial pressure, thus, it plays a central role in the maintenance and regulation of cardiovascular homeostasis (de Bold et al., 1981; de Bold, 1985; Brenner et al., 1990; Anand-Srivastava and Trachte, 1993; Pandey, 2005, 2011). In addition to its natriuretic, diuretic, vasorelaxant, antimitogenic, antihypertrophic, and anti-inflammatory activities, ANP inhibits the release of renin from the kidneys, aldosterone from the adrenal glands, vasopressin from posterior pituitary, and progesterone from Leydig tumor (MA-10) cells, while stimulating the synthesis and release of testosterone from normal Leydig cells in the testes, progesterone from granulosa-leuteal cells, and luteinizing hormone from anterior pituitary gland (Inagami, 1989; Brenner et al., 1990; Levin et al., 1998; Pandey, 2005). A number of studies have documented that ANP has always been found to increase the intracellular accumulation of cGMP, however, to decrease the levels of cAMP; Ca^2+^, and inositol triphosphate (IP_3_) in agonist hormone-treated cells and tissues (Waldman et al., 1984; Pandey et al., 1985, 1988; Khurana and Pandey, 1993, 1996; Pandey, 2005; Turovsky et al., 2013). It has also been suggested that ANP decreases the cAMP levels by stimulating the cGMP-specific phosphodiesterases; however, in certain cells and tissue types, ANP did not decrease or change the cAMP concentrations. Several studies have indicated that ANP diminishes the Ca^2+^ signals probably by activating the Ca^2+^ extrusion processes by protein kinase-G (PKG) specifically in endothelial and vascular smooth muscle cells (VSMCs; Rashatwar et al., 1987; Zolle et al., 2000; Pandey, 2005).

Among the natriuretic peptides (NPs) hormone family, ANP is the first described member, later, two other members of NP family; brain natriuretic peptide (BNP) and C-type natriuretic peptide (CNP) were identified and characterized, which also exhibited biochemical and structural properties similar to ANP; however, each prepro NP hormone is encoded from a separate gene (Rosenzweig and Seidman, 1991; Levin et al., 1998). Although, all three NPs (ANP, BNP, and CNP) have highly homologous structure, they bind to specific NP receptors and elicit discrete biological and physiological functions (Brenner et al., 1990). There have been three subtypes of NP receptors, namely; NP receptor-A, NP receptor-B, and NP peptide receptor-C (NPRA, NPRB, and NPRC, respectively), which were identified and characterized by molecular cloning (Pandey and Singh, 1990; Garbers, 1992). Interestingly, both ANP and BNP bind to NPRA, which produces the intracellular second messenger cGMP; however, CNP binds NPRB, which also generates cGMP, but all three NPs indiscriminately bind and activate NPRC, which lacks GC catalytic domain (Fuller et al., 1988; Drewett and Garbers, 1994; Lucas et al., 2000; Sharma, 2002; Pandey, 2005). The cellular, biochemical, and pharmacological aspects of NPs and their cognate receptors have revealed classic hallmark of physiological and pathophysiological functional significance, including; renal, cardiac, vascular, neuronal, and immunological aspects in health and disease (Pandey, 2005, 2011; Kishimoto et al., 2011). It has been suggested that ANP suppresses Na^+^-reabsorption at the collecting duct of the kidneys, inhibits renin synthesis and release, and stimulates natriuresis and diuresis, thereby, lowers blood pressure and blood volume and maintains cardiovascular homeostasis (de Bold, 1985; Brenner et al., 1990; Levin et al., 1998; Pandey, 2005). In the vasculature, ANP relaxes VSMCs thus causing the immediate vasorelaxant effect in the vascular bed (Levin et al., 1998; Pandey, 2005).

The expression and activity of NPRA is regulated by various hormonal agents, including its ligand ANP (Pandey, 1993, 2005; Cao et al., 1995; Pandey et al., 2002). The studies detailing the *Npr1* (coding for NPRA) gene-disruption in mice have revealed the functional significance of NPRA in the control of blood pressure and cardiovascular disease states (Oliver et al., 1997; Shi et al., 2001; Holtwick et al., 2002; Vellaichamy et al., 2005; Kishimoto et al., 2011; Pandey, 2011; Yoshihara et al., 2014). Mice lacking NPRA develop high blood pressure and severe cardiac hypertrophy, fibrosis, and disorders that are reminiscent of heart disease as seen in untreated human hypertensive patients (Vellaichamy et al., 2007, 2014; Zhao et al., 2013). The regulated expression of CNP is derived from endothelial cells, which targets NPRB on the adjacent smooth muscle cells (Suga et al., 1992). Thus, the principal role of CNP is considered as a direct vasodilator involved in the regulation of vascular tone through activation of GC-B/NPRB on smooth muscle cells in the vascular beds (Hama et al., 1994). The objective of this current review is to summarize and document the findings and discoveries with particular emphasis of cellular signaling and physiological and pathological significance of ANP/NPRA in relation to the increased production of intracellular second messenger cGMP and inhibition of the phosphoinositide (IP_3_) hydrolysis, Ca^2+^ release, and protein kinase C (PKC) activity in target cells.

## HISTORICAL BACKGROUND

Thirty-three years ago, the pioneer discovery by de Bold and his coworkers established that atrial extracts contained natriuretic and diuretic activity which led to the isolation and nomenclature of ANF, usually referred to as ANP (de Bold et al., 1981; de Bold, 1985). Now, it is considered that ANP is primarily synthesized and secreted in the granules of heart atrium and BNP is largely synthesized in the heart ventricle and displays most variability in the primary structure. Although, the atrium is the primary site of synthesis for ANP, however, ventricle also produces ANP but at the levels of 100-fold to 1000-fold lower than that of the atrium, respectively (Kojima et al., 1989). CNP was isolated from the porcine brain, however, is mostly present in the endothelial cells of the vasculature and is highly conserved among the mammalian species (Rosenzweig and Seidman, 1991). The primary structure deduced from the cDNA synthesis, suggested that ANP is synthesized first as the 152-amino acid prepro-ANP molecule that contains sequences of active peptide in its carboxyl-terminal region (Maki et al., 1984). The biologically active ANP is released by proteolytic cleavage of pro-ANP molecule into predominantly 28-amino acid active (residues 99–126) and the 98 amino acid inactive (residues 1–98) molecules. The active form of ANP has a disulfide-bonded loop between cysteine 105 and 121, which seems to be essential for the biological activity (Brenner et al., 1990). Initially, different lengths of sequences of ANP were identified and synthesized for the studies of structure-activity relationship, and it was suggested that the ring structure of ANP with a disulfide-bonded loop is essential for its biological activities (Rosenzweig and Seidman, 1991). All three NPs contain highly conserved amino acid sequences with a 17-residues disulfide-bonded ring but deviate from each other in the N-terminal and C-terminal flanking amino acid sequences. Furthermore, the C-terminal sequence extending from the ring structure to Asn-Phe-Arg-Tyr is essential for the biological activity of ANP. The amino acid sequence of ANP is almost identical across the mammalian species, except at the position 10, which is substituted with isoleucine in rat, mouse, and rabbit, however, in human, dog, and bovine, ANPs have methionine in this position (Misono et al., 1984). Subsequently, BNP and CNP were both isolated and characterized from the porcine brain extracts (Sudoh et al., 1988, 1990). BNP is predominantly synthesized and secreted from the heart ventricle (Phillips et al., 1991). Similarly, CNP is predominantly localized in the central nervous system and endothelial cells and is considered as a non-circulatory peptide hormone (Suga et al., 1992).

## NATRIURETIC PEPTIDES SYNTHESIS AND SECRETION

It has been suggested that the processing of preprohormone to prohormone molecule and the cleavage and secretion of biologically active mature 28-residue ANP molecule occurs predominantly in response to atrial distension (de Bold, 1985; Brenner et al., 1990). Usually, ANP concentration ranges from 50 to 100-fold higher than BNP; however, the expression of both ANP and BNP increases dramatically in the atrium and ventricle in the condition of cardiac disorders and heart failure (Mukoyama et al., 1991). During the disease states, the ventricle becomes the primary site of synthesis and release for BNP. In congestive heart failure (CHF) patients, the concentrations of both ANP and BNP increase greater than the control values, however, the BNP concentration increases 10-fold to 50-fold higher than a comparative increases in the ANP levels (Mukoyama et al., 1991). Those previous findings indicated that ANP and BNP elicit distinct physiological and pathophysiological effects, nevertheless, both hormones show similar hemodynamic responses, but BNP exerts a longer duration of action and causes enhanced natriuretic responses as compared with ANP (Yoshimura et al., 1991; Omland et al., 1996). It has been suggested that the cardiac atrium expresses almost 50-fold to 100-fold or even higher levels of ANP mRNA as compared with extra -cardiac tissues (Gardner et al., 1986). Interestingly, higher ventricular ANP levels have been found in the developing embryos and fetuses; however, both mRNA and peptide levels of ANP decline rapidly during the prenatal period (Cameron et al., 1996). On the other hand, CNP does not seem to behave as a cardiac hormone and its concentration is extremely low in the circulation (Igaki et al., 1996). It is believed that CNP is largely localized in the central nervous system and in the vascular endothelial cells (Ogawa et al., 1992; Suga et al., 1992, 1993; Tamura et al., 1996; Chen and Burnett, 1998). Another class of NPs is the D-type natriuretic peptide (DNP) that represents an additional member in the NP hormone family and is largely present in the venom of the green mamba (*Dendroaspis angusticeps*) as a 38-amino acid peptide molecule (Schweitz et al., 1992; Lisy et al., 1999). In addition, a 32-amino acid peptide termed as urodilatin (URO) is identical to C-terminal sequence of pro-ANP, which is largely present only in the urine (Schulz-Knappe et al., 1988). Initially, URO was purified from the human urine and is considered to be synthesized only in the kidneys (Saxenhofer et al., 1990). The immunohistochemical staining indicated that URO is largely present in the cortical tubules around the collecting ducts of the kidneys (Meyer et al., 1996; Doust et al., 2005).

In the circulation, the half-life of BNP is greater than ANP, thus the evaluation of the diagnostic importance of the NPs have mostly favored BNP. The inactive N-terminal fragment of BNP (NT-proBNP) has even a greater half-life than the BNP. The plasma levels of both BNP and NT-proBNP are markedly elevated under the pathophysiological conditions of cardiac dysfunction, including diastolic dysfunction, CHF, and pulmonary embolism (Felker et al., 2006; Jaffe et al., 2006). The basal plasma levels of BNP vary from 5 to 50 pg/ml and NT-proBNP levels range from 10 to 150 pg/ml. An abnormal range is considered as 100 pg/ml for BNP and 125 pg/ml for NT-proBNP (Felker et al., 2006). Nevertheless, the secretion of both ANP and BNP from the ventricular myocytes increases proportionally in relation to the magnitude of cardiac dysfunction or heart failure condition (Yoshimura et al., 1993). It has been suggested that BNP acts as an important prognostic indicator in the CHF patients, however, NT-proBNP is considered to be a stronger risk bio-indicator for cardiovascular events (Doust et al., 2005). Both BNP and NT-proBNP seem to provide an ideal tool to be utilized as blood tests to diagnose cardiac disorders in patients with high risk of heart failure, diabetes, chronic kidney disease, and coronary artery disease (Khan et al., 2006; Freestone et al., 2008; Czucz et al., 2011; Ganem et al., 2011). The BNP level is increased to almost 200 pg/ml and NT-proBNP levels reaches to approximately 1200 pg/ml in patients with reduced creatinin clearance (McCullough et al., 2003; Anwaruddin et al., 2006).

## IDENTIFICATION AND CHARACTERIZATION OF RECEPTOR MEMBRANE GUANYLYL CYCLASE

The previous studies using cross-linking and photoaffinity labeling procedures, have shown the existence of NP receptors with a wide range of molecular weight (M_r_) of the 60–180 kDa (Misono et al., 1985; Schenk et al., 1985; Vandlen et al., 1985; Meloche et al., 1986; Pandey et al., 1986). Initially, NP receptors were identified with varying receptor density in different cells and tissue types (**Table 1**). Subsequently, high affinity ANP binding sites were with GC activity were co-purified (Kuno et al., 1986; Paul et al., 1987; Takayanagi et al., 1987; Meloche et al., 1988) On the basis of biological activity of different ANP analogs, NP receptors were classified and characterized as biologically active and clearance or silent receptors (Maack et al., 1987). Subsequently, three distinct subtypes of NP receptors were identified, which appeared to be specific to different cells and tissues (Pandey et al., 1988). Based on the cellular, biochemical, and molecular biological studies, the NPs and their receptors are quite widespread in cell and tissue distributions (Leitman et al., 1988; Pandey et al., 1988; Brenner et al., 1990; Marala et al., 1992; Levin et al., 1998; Pandey, 2005). Molecular cloning and expression of cDNA from mouse, rate, and human, led to identify and characterize the primary structure of three distinct subtypes of NP receptors, which are currently designated as GC-A/NPRA, GC-B/NPRB, and NPRC (Fuller et al., 1988; Chinkers et al., 1989; Schulz et al., 1989; Pandey and Singh, 1990; Duda et al., 1991). The general topological structures of GC-A/NPRA and GC-B/NPRB are consistent with at least four distinct domains, including extracellular ligand-binding domain, a single transmembrane spanning region, and intracellular protein kinase-like homology domain (protein-KHD), and GC catalytic domain. The transmembrane GC receptors contain a single cyclase catalytic site per protein molecule, however, based on the structural modeling data two polypeptide chains seem to be required to activate GC-A/NPRA (Wilson and Chinkers, 1995; Labrecque et al., 1999; van den Akker et al., 2000). It has been indicated that the dimerization region of the receptor is located between the KHD and GC catalytic domain that has been predicted to form an amphipathic alpha helix structure. The GC-B/NPRB has the overall domain structure similar to that of GC-A/NPRA with binding affinity to CNP also produces the intracellular second messenger cGMP (Schulz et al., 1989; Koller et al., 1991; Lucas et al., 2000). NPRA is considered as the dominant subtype of the NP receptors found in peripheral organs and mediates most of the known functions of ANP and BNP hormones. Nevertheless, NPRB is localized mainly in the central nervous system and vascular tissues, which is thought to mediate the actions of CNP in the brain and also in the vascular bed. There are increasing numbers of other GC receptors; however, the specific ligands for these receptors are still being identified (**Table 2**). The third member of the NP receptor family, NPRC, constitutes a large extracellular domain of 496-amino acids, a single transmembrane domain, and a very short 37-amino acid cytoplasmic tail that contains no sequence homology with any other known membrane receptor proteins and has been given the name by default as clearance receptor (Fuller et al., 1988). The extracellular region of NPRC is approximately 30% identical to both GC-A/NPRA and GC-B/NPRB. Studies using the ligand receptor binding as a criterion, have shown that NPRC has much less stringent specificity and affinity for structural variants of ANP than does NPRA (Bovy, 1990). The extracellular domain of NPRC possesses two pairs of cysteine residues along with one isolated cysteine near the transmembrane domain of the receptor. However, three potential signals for N-glycosylation and several serine and threonine for O-linked glycosylation sites are known to be present in the extracellular domain of NPRC (Fuller et al., 1988). Previously, it has been suggested that NPRC may function as a clearance receptor to remove and clear NPs from the circulation, however, a number of studies have provided the evidence that NPRC plays roles in the biological actions of NPs (Anand-Srivastava and Trachte, 1993; Matsukawa et al., 1999; Zhou and Murthy, 2003). Thus, it is evident that the clearance name carries only by a default nomenclature to NPRC.

**Table 1:** ANP-dependent binding parameters of GC-A/NPRA and intracellular accumulation of cGMP in different cell types.

Cell type	ANP-dependent Intracellular cGMP (fold stimulation)	Ligand binding parameters of NPRA	
		kd value (Molar)	*B*_max_ (receptor site/cell)
Endothelial cells	15	10–100 pM	0.5 × 10^5^
Granulosa cells	30	10–100 pM	0.5 × 10^5^
Glomerulosa cells	50	100–1 pM	2 × 10^5^
MA-10 cells	1,500	100–1 nM	1 × 10^6^
MDCK cells	50	10–100 pM	0.5 × 10^5^
N4TG1 cells	30	1–100 pM	0.5 × 10^5^
Primary Ledig cells	60	10–100 pM	0. 5 × 10^5^
RTASM cells	10	1–100 pM	0.2 × 10^5^

*HEK-293 cells, human embryonic Kidney-293 cells; kd, dissociation constant; *B*_max_, receptor density; MA-10 cells, Leydig tumor cells; MDCK cells, Maiden-Darby kidney epithelial cells; N4TG1 cells, neuroblastoma cells; RTASM, rat thoracic aortic smooth muscle cells.*

**Table 2:** The distribution of natriuretic peptide receptors (NPRA, NPRB, and NPRC) and their gene-knockout phenotype.

Receptor	Ligand	Tissue-specific distribution	Cell-specific distribution	Gene-knockout phenotype
NPRA *(Npr1)*	ANP/BNP	Kidney, adrenal glands, brain, heart, liver, lung, olfactory, ovary, pituitary gland, placenta, testis, thymus, vascular beds, liver, ileum	Renal epithelial and mesangial cells, vascular smooth muscle cells, endothelial cells, Leydig cells, granulosa cells, fibroblasts, Neuroblastoma, LLCPk-1, MDCK cells	High blood pressure, hypertension, cardiac hypertrophy and fibrosis, inflammation, volume overload, reduced testosterone
NPRB *(Npr2)*	CNP	Adrenal glands, brain, cartilage, fibroblast, heart, lung, ovary, pituitary gland, placenta, testis, thymus, vascular beds	Vascular smooth muscle cells, fibroblasts, chondrocytes	Dwarfism, decreased adiposity, female sterility, seizures, vascular complication
NPRC *(Npr3)*	ANP, BNP, CNP	Kidney, heart, brain liver, vascular bed, intestine	Vascular smooth muscle cells, endothelial cells, mesangial cells, fibroblasts	Bone deformation, skeletal over-growth, long bone overgrowth
GC-D	Guanylyn/uroguanylyn	Olfactory neuroepithelium		
GC-E/(ROS-GC-1)	Ca^2+^-binding proteins	Retina, pineal gland		
GC-F/(ROS-GC-2)	Ca^2+^-binding proteins	Retina, rod outer segment		
GC-G	Orphan	Skeletal muscle, lung, intestine, and kidney		
GC-Y-X1	Orphan	Sensory neurons of *C*. *elegans*		

*NPRA, natriuretic peptide receptor-A; *Npr1*, coding for guanylyl cyclase/natriuretic peptide receptor-A; NPRB, natriuretic peptide receptor-B; *Npr2*, coding for guanylyl cyclase/natriuretic peptide receptor-B; NPRC, natriuretic peptide receptor-C; *Npr3*, coding for natriuretic peptide clearance receptor.*

## INTERNALIZATION AND DOWN-REGULATION OF GC-A/NPRA

The ligand-dependent internalization plays important role in the receptor down-regulation and signaling process. Down-regulation of GC-A/NPRA has been reported in a number of cells, including PC-12 cells containing endogenous receptors and transfected COS-7 and HEK-293 cells harboring recombinant receptors (Rathinavelu and Isom, 1991; Pandey, 1993; Pandey et al., 2000a, 2002). The carboxyl-terminal deletion mutation has shown that the specific sites in the GC catalytic domain and KHD of NPRA, play critical roles in the endocytosis and sequestration of the receptor (Pandey et al., 2000a). Previous studies have also indicated that after prolonged treatment of cultured cells with ANP, both the receptor density and GC activity were decreased with simultaneous reduction in mRNA of levels NPRA (Fujio et al., 1994; Cao et al., 1995, 1998; Hum et al., 2004). In addition, transforming growth factor-β1 (TGF-β1), angiotensin II (ANG II), and endothelin (ET-1) have also been shown to reduce mRNA levels of NPRA in various types of cultured cells (Fujio et al., 1994; Chen and Gardner, 2003; Garg and Pandey, 2003; Arise and Pandey, 2006). Those previous studies demonstrated that a decrease in mRNA levels of GC-A/NPRA correlated with the repressed transcriptional activity of the receptor. On the other hand, mRNA levels of NPRA are greatly increased by retinoic acid and histonedeacetylase inhibitor treatments (Kumar et al., 2010, 2014a,b). It has been suggested that NPRA exists in the phosphorylated state and the addition of ANP causes a decrease in the phosphate contents as well as reduction or desensitization of the ANP-dependent GC catalytic activity of NPRA Potter and Garbers, 1992). The apparent mechanism of desensitization of NPRA is in contrast to many other cell-surface hormone receptors, which appear to be desensitized by phosphorylation (Sibley et al., 1987; Hugnir and Greengard, 1990; Lefkowitz et al., 1998; Sorkin and von Zastrow, 2002). The initial findings have also indicated that ANP stimulates phosphorylation of NPRA (Ballerman et al., 1988; Pandey, 1989; Duda and Sharma, 1990; Larose et al., 1992). Later, it was suggested that cGMP-dependent PKG, a serine/threonine kinase is also phosphorylates NPRA (Airhart et al., 2003).

The down-regulation of NPRC also seems to be associated with increased internalization of the ligand-receptor complexes involving receptor-mediated endocytosis and trafficking mechanisms of this receptor protein (Pandey, 1992). The phenomenon of down-regulation of NPRC has been largely documented in cultured VSMCs, which predominantly contain a high density of NPRC (Neuser and Bellermann, 1986; Hirata et al., 1987; Hughes et al., 1987; Pandey, 1992; Anand-Srivastava, 2000). The metabolic processing of ANP involving NPRC has been reported by several investigators utilizing VSMCs (Hirata et al., 1985; Napier et al., 1986; Murthy et al., 1989; Nussenzveig et al., 1990; Pandey, 1992, 2005; Cohen et al., 1996; Anand-Srivastava, 2000). It has been suggested that a population of the internalized NPRC also recycles back to the plasma membrane (Pandey, 1992).

## ACTIVATION OF GC-A/NPRA GENERATES INTRACELLULAR SECOND MESSENGER cGMP

It is believed that cGMP is generated as a result of ANP binding to the extracellular domain of GC-A/NPRA, which probably allosterically regulates an increased activity of the receptor protein (Pandey, 1993, 2005, 2011; Drewett and Garbers, 1994; Pandey et al., 2002; Sharma, 2002; Sharma and Duda, 2010). The initial findings showed that ANP markedly increases cGMP in target tissues in a dose-related manner (Hamet et al., 1984; Waldman et al., 1984; Pandey et al., 1985). Previous studies have also indicated that the binding of ANP to GC-A/NPRA by itself is probably not sufficient to stimulate GC catalytic activity and the production of cGMP, however, it requires ATP (Kurose et al., 1987; Chinkers et al., 1991; Goraczniak et al., 1992). Because the non-hydrolyzable analogs of ATP mimicked ANP effect, it was suggested that ATP acts directly by allosteric regulation of GC catalytic activity of NPRA. Both the ligand binding and the interaction of ATP with the KHD of the receptor increase the cGMP production without affecting the affinity for the substrate (Kurose et al., 1987; Chang et al., 1990; Duda et al., 1991). Molecular cloning and overexpression of NPRA demonstrated that GC catalytic domain cannot be activated by ANP alone without ATP-binding to KHD region of the receptor (Chinkers et al., 1991; Larose et al., 1991; Wong et al., 1995). Further studies provided the evidence that ATP binding to KHD of NPRA is important for the effectors coupling of GC family of receptors (Goraczniak et al., 1992; Sharma, 2002).

Deletion of the KHD of GC-A/NPRA and GC-B/NPRB has been suggested that KHD represses the GC catalytic activity of these receptors (Chinkers et al., 1989). At the same time, another model was proposed indicating that KHD was not a repressor; however, ATP was required to activate the catalytic domain of NPRA (Goraczniak et al., 1992; Sharma, 2002). Both NPRA and NPRB contain a glycine-rich ATP binding motif within the KHD, which is known as glycine-rich consensus sequence (Duda et al., 1991, 1993; Goraczniak et al., 1992). The juxtamembrane hinge structure of NPRA undergoes a significant conformational change in response to ligand binding, and it may play an important role in transmembrane signaling process (Huo et al., 1999). The amino acid sequence near the transmembrane region is well conserved in GC-A/NPRA that contains several closely located proline residues and a pair of cysteine residues. The mutation of one of the proline in this region renders the receptor to bind the ligand but blocks GC catalytic activity (Huo et al., 1999). Similarly, in the juxtamembrane hinge region, the elimination of disulfide bond of cysteine residues resulted in constitutive activation of NPRA. Those previous findings suggested that juxtamembrane hinge region of NPRA may play a critical role in receptor activation and signal transduction mechanisms of GC-coupled receptors.

The glycosylation of the receptor seems to be essential for ligand binding activity of GC-A/NPRA (Lowe and Fendly, 1992; Fenrick et al., 1997). However, it has also been suggested that glycosylation may not be required for ligand binding of NPRA (Miyagi et al., 2000). The mutational analyses of N-linked glycosylation consensus sites in guanylyl cyclase-C (GC-C) have indicated that certain amino acid residues might be important for receptor stability (Hesegawa et al., 1999). The glycosylation sites onto the GC-A/NPRA binding domain have been found to be scattered on the surface of the receptor with the exception of the hormone binding site and dimer interface (van den Akker, 2001). The glycosylation sites have been implicated to function in proper folding and stability of NPRA (Lowe and Fendly, 1992; Koller et al., 1993; Heim et al., 1996). Nevertheless, the glycosylation of the extracellular domain of NPRA can be considered of significant importance for receptor orientation and packaging on the cell surface similar to that of other plasma membrane receptor proteins (Wormald and Dwek, 1999). Nevertheless, it should be noted that there is no appreciable conservation of the precise position of the glycosylation sites within the members of GC-receptor family. Clearly, more studies are needed to confirm the functional roles of glycosylation in the transmembrane signaling processes of both GC-A/NPRA and GC-B/NPRB protein molecules.

## ANP/NPRA SIGNALING INHIBITS PHOSPHOINOSITIDE HYDROLYSIS, Ca^2+^ RELEASE, AND PKC ACTIVITY

Previous studies have demonstrated that ANP significantly decreased the hydrolysis of phosphoinositide in murine Leydig tumor (MA-10) cells in a dose-dependent manner and the H-8, a specific inhibitor of PKG, reversed the inhibitory effect of ANP on the generation of inositol phosphates, supporting the involvement of PKG in this process (Khurana and Pandey, 1995). ANP has also been shown to inhibit both autophosphorylation and enzymatic activity of PKC in different cell systems (Pandey, 1989, 1994a,b; Kumar et al., 1997). It is not yet clear if the ANP-dependent inhibitory effects on the phosphoinositide metabolism and PKC autophosphorylation and/or enzyme activity are exerted in a composite manner to negatively regulate the phosphoinositide, Ca^2+^, and PKC involving ANP/NPRA/cGMP/PKG cascade. It is also possible that the effect of ANP is transmitted to block the IP_3_ and Ca^2+^ signaling pathways independently in response to particular agonist stimulation. It has been suggested that potassium channels can be stimulated by ANP through the activation of PKGs, which require ATP and G-proteins (White et al., 1993). However, the possible involvements of potassium channels in the ANP-dependent inhibitory responses on the generation of inositol phosphates are not yet clearly understood. ANP has also been shown to stimulate the formation of inositol phosphates in cultured VSMCs, however, in the inner medullary collecting duct cells and smooth muscle tissues, ANP stimulated the production of inositol phosphates at lower dosages, and inhibited the formation of these metabolites at higher dosages, which increase intracellular generation of cGMP (Resink et al., 1988; Hirata et al., 1989; Teitelbaum et al., 1990; Berl et al., 1991). Thus the heterogeneity of NP receptors and their diverse cellular distribution suggest that different mechanisms might be involved in the cellular action of ANP/NPRA/cGMP (Anand-Srivastava and Trachte, 1993; Pandey, 2001, 2002, 2011). It has also been shown that ANP inhibits the thrombin-induced synthesis and release of endothelin in cultured rat aortic endothelial cells by blocking the phosphoinositide breakdown (Emori et al., 1993).

**FIGURE 1:**
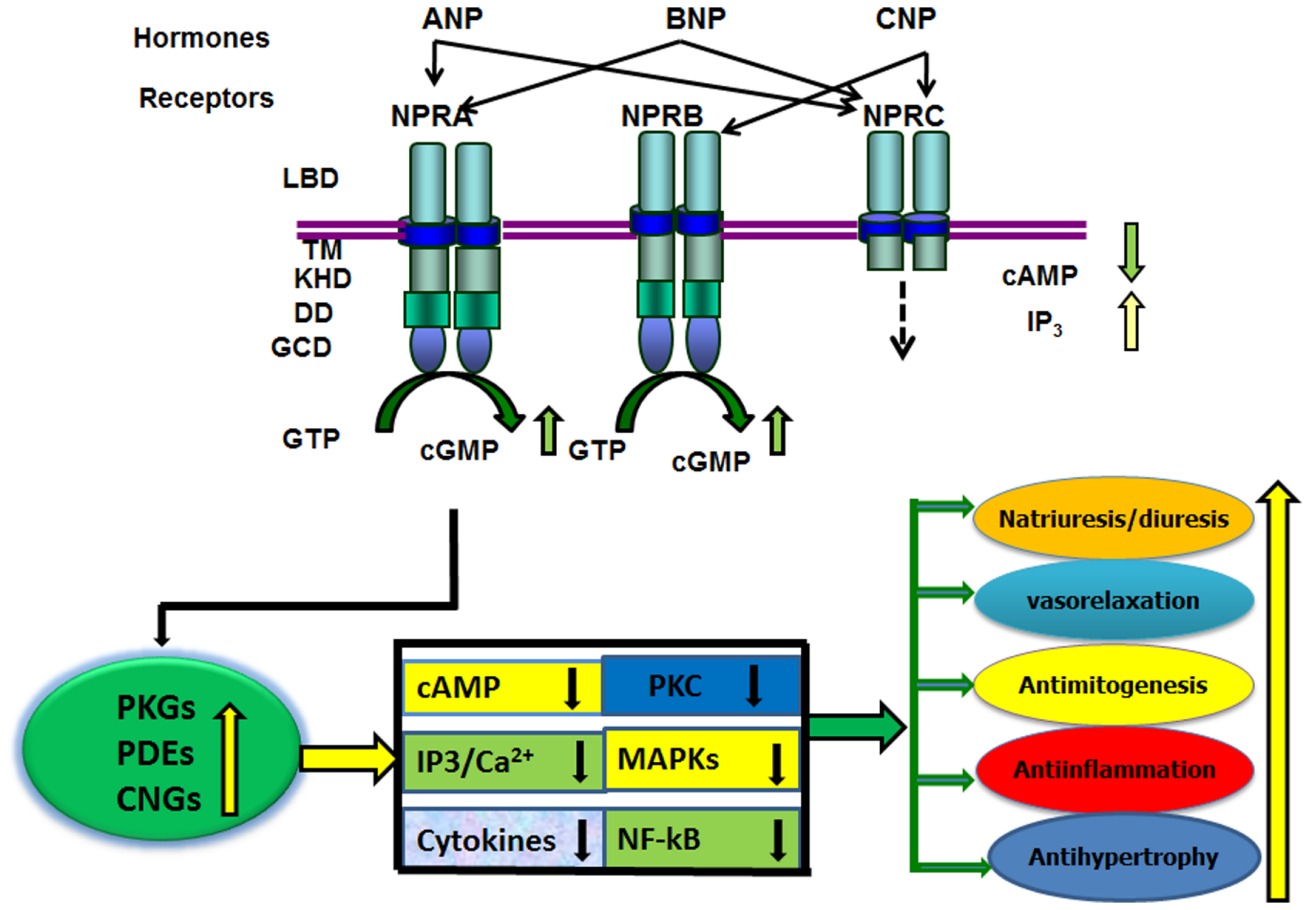
**Diagram represents the ligand specificity and physiological function(s) of GC-A/NPRA.** The ligand-binding to NPRA generates second messenger cGMP from the hydrolysis of GTP. An increased level of intracellular cGMP is produced, which activates three known cGMP effecter molecules namely; cGMP-dependent protein kinases (PKGs), cGMP-dependent phosphodiesterases (PDEs), and cGMP-dependent ion-gated channels (CNGs). The ANP/NPRA/cGMP signaling may antagonize a number of pathways including; intracellular formation of cAMP, Ca^2+^, IP_3;_ cytokine expression; and the activation of protein kinase C (PKC) and mitogen-activated protein kinases (MAPKs). The resulting signaling cascade can mimic the physiological responses of ANP/NPRA. LBD, ligand binding domain; TM transmembrane region; protein-KHD, protein kinase-like homology domain; and GCD, guanylyl cyclase catalytic domain; DD, dimerization domain of NPRA and NPRB. The ligand binding region, transmembrane domain, and small intracellular tail region of NPRC are indicated.

In addition to the stimulatory effect of ANP on GC activity, it has also been shown to reduce adenylyl cyclase and phospholipase C activities, sodium influx, and Ca^2+^ concentrations (Brenner et al., 1990; Anand-Srivastava and Trachte, 1993; Pandey, 2005). The increased production of cGMP in response to ANP correlates with the effects of dibutyryl-cGMP. The most compelling evidence supporting a role for cGMP effects was obtained with selective NPRA antagonists, A71915 and HS-121-1 in the kidneys (von Geldern et al., 1990; Sano et al., 1992). Those previous studies established that ANP effect is largely mediated by cGMP through the activation of GC-A/NPRA. In general, evidence suggests that biological activity of ANP/NPRA enhances the generation of the intracellular second messenger cGMP and decreases the levels of cAMP, Ca^2+^, and IP_3_ along with the antagonistic effects on PKC and mitogen-activated protein kinases (MAPKs) in target cells (**Figure 1**). ANP has been reported to induce cGMP-dependent acrosomal reaction in both capacitated and non-capacitated spermatozoa (Anderson et al., 1994). Furthermore, the acrosome reaction was essentially equal in magnitude when induced with ANP or Ca^2+^ ionophore A23187. However, higher concentrations of ANP were required to induce acrosomal reaction in capacitated as compared with non-capacitated spermatozoa. Those previous findings indicated that ANP-induced human acrosomal reaction does not require physiological concentrations of extracellular Ca^2+^. Acrosomal reaction is known to involve various extracellular signals, including cAMP (Anderson et al., 1992), cGMP (Komatsu et al., 1990), prostaglandins, Ca^2+^ and IP_3_ (Thomas and Meizel, 1989), and diacylglycerol (Breitbart et al., 1992).

The established biochemical and cellular effects of ANP in the adrenal glomerulosa cells showed the activation of GC activity and K^+^ channel conductance; whereas T-type Ca^2+^ channels conductance and adenylyl cyclase activity are suppressed (Anand-Srivastava and Trachte, 1993). The correlative evidence between ANP-induced cGMP accumulation and vasodilation has suggested the role of cGMP as the intracellular second messenger of dilator responses to ANP (Brenner et al., 1990; Anand-Srivastava and Trachte, 1993; Cao et al., 1995; Pandey, 2005). ANP as well as cGMP analogs have been found to reduce the agonist-induced increases in cytosolic Ca^2+^ concentrations (Hassid, 1986; Lincoln et al., 1994; Pandey, 2005). It has been suggested that cGMP activates sarcolemmal Ca^2+^-ATPase, and this mechanism seems to be important in the ANP-induced decreases in cytosolic Ca^2+^ in VSMCs (Rashatwar et al., 1987; Cornwell and Lincoln, 1989; Levin et al., 1998; Pandey, 2005). Nevertheless, it is anticipated that the ultimate effect of ANP in VSMCs could be due to production of cGMP and the activation of PKG (Lincoln et al., 1994; Kumar et al., 1997). However, more studies are needed to define the biochemical and molecular basis of NP actions in vasculature, including VSMCs and endothelial cells.

Initial studies from our laboratory and data published from others have also shown that both ANP and cGMP inhibited the autophosphorylation and enzymatic activity of PKC in the plasma membrane preparations of various target cells (Rogers et al., 1988; Sauro and Fitzpatrick, 1990; Pandey, 1994a,b; Kumar et al., 1997). The activation of PKC triggers the agonist-dependent phosphorylation and activity of numerous cellular proteins causing alteration in many physiological and pathophysiological conditions, including hypertension, cardiac hypertrophy, ischemia, atherosclerosis, stroke, and neurological disorders (Louis et al., 1988; Turla et al., 1990; Komuro et al., 1991; Kumar et al., 1997). PKC is believed to be a multigene family, consisting of at least 12 isoenzymes that can be classified into classical, novel, and atypical forms (Hug and Sarre, 1993; Dekker and Parker, 1994). These PKC isoenzymes are multifunctional serine/threonine kinases that are largely activated by Ca^2+^/phospholipids and phorbol esters. However, some of these isoforms (e, δ, ή, and φ) do not require Ca^2+^, while other isoforms (ζ and 𝜀) do not require Ca^2+^ or phospholipid for PKC enzymatic activity. Previous studies have indicated that vasoconstrictive agents, including ANG II and ET-1, were able to activate several-fold PKC activity in cultured VSMCs, however, ANP potently antagonized the ANG II- and ET-1-stimulated PKC activity in the ANP/NPRA-dependent manner (Kumar et al., 1997; Pandey, 2005). The inhibitory effect of ANP was greatly amplified if cell were transfected with both PKC-α and NPRA cDNAs. The pretreatment of cells with NPRA antagonist A-71915, significantly blocked the production of cGMP as well as the inhibitory effect of ANP on PKC activity (Kumar et al., 1997). The results of those previous studies provided strong evidence that ANP antagonizes the PKC activation involving ANP/NPRA/cGMP signaling cascade. Agonists that activate PKC also produce two distinct second messengers, IP3, which activates cytosolic free Ca^2+^ and diacylglycesol, which stimulates PKC activity (Berridge and Irvine, 1989; Exton, 1990; Rasmussen et al., 1995; Kumar et al., 1997). Our previous studies have suggested that ANP inhibits the formation of IP3 in a cGMP-dependent manner in the intact cells, suggesting that the inhibitory effect of ANP on PKC activity might be linked with its antagonistic action on IP_3_ formation, however, more studies are needed to support these observations in various ANP-responsive cell and tissues systems.

## EFFECT OF NPRA ON THE INHIBITION OF MAPKs ACTIVITY AND CELL PROLIFERATION

It has been shown that cGMP analogs mimicked the antiproliferative action of ANP, indicating that it exerts the antimitogenic effects largely through the intracellular second messenger cGMP (Lincoln et al., 1994; Hutchinson et al., 1997; Pandey et al., 2000b; Sharma et al., 2002). ANP has been shown to inhibit collagen synthesis in cardiac fibroblasts and also it inhibits hypertrophy of cardiac myocytes (Calderone et al., 1998; Masciotra et al., 1999; Silberbach et al., 1999; Horio et al., 2000; Gopi et al., 2013). Similarly, PKG has been shown to suppress extracellular matrix production in VSMCs (Dey et al., 1998). Both NPRA and NPRC, have been suggested to play a role in ANP-dependent antimitogenic responses (Prins et al., 1996; Hutchinson et al., 1997; Pandey et al., 2000b; Sharma et al., 2002; Tripathi and Pandey, 2012). ANP has been shown to act as a growth suppressor in a variety of cell types including; kidney, heart, neurons, thymus, vasculature, and fibroblasts (Levin et al., 1998; Pandey, 2005). Previous studies have demonstrated that ANP inhibits ANGII- and platelet-derived growth factor (PDGF) -dependent MAPK activity in different tissues and cell types (Sugimoto et al., 1993; Prins et al., 1996; Pandey et al., 2000b; Sharma et al., 2002; Tripathi and Pandey, 2012). However, in astroglial cells, ANP was shown to inhibit extracellular-regulated MAPK (Erk1/2) activity through NPRC (Prins et al., 1996). In contrast, recent findings have indicated that des- (Cys^105^–Cys^121^) -ANP, a ligand selective to NPRC, did not inhibit basal or serum-stimulated MAPK, however, CNP, which acts through NPRB, potently inhibited MAPK activity in fibroblasts in a cGMP-dependent manner (Chrisman and Garbers, 1999).

It has been postulated that cGMP-dependent signaling mechanisms of GC-A/NPRA are initiated probably at the level of gene transcription; however, the exact mechanism of this activation remains to be elucidated. A previous report also indicated that cGMP/PKG signaling was able to increase the MAPK activity in contractile rat VSMCs (Komalavilas et al., 1999). However, the process by which cGMP/PKG leads to the activation of MAPKs is unclear. Similarly, cAMP- and PKG have also been shown to inhibit as well as to activate MAPKs pathways, depending on the cell types and culture conditions (Bornfildt and Krebs, 1999). However, the involvement of specific ANP receptor subtypes in the inhibitory effects of ANP on the agonist-stimulated MAPKs activity is controversial. Indeed, more studies are needed to establish the underlying mechanisms of the antiproliferative effect of ANP in target cells. ANP has also been shown to induce apoptosis in cultured VSMCs and in neonatal rat cardiac myocytes (Trindade et al., 1995; Wu et al., 1997). The apoptotic effect of ANP was mimicked by 8-bromo-cGMP, a membrane-permeable analog of cGMP, and also by nitroprusside, an activator of soluble guanylyl cyclase. Furthermore, the effect of ANP was greatly potentiated by a cGMP-specific phosphodiesterase inhibitor zaprinast. It has been indicated that norepinephrine, a myocyte growth and proliferative effector molecule, inhibited ANP-induced apoptosis via activation of β-adrenergic receptor and elevation of cAMP (Wu et al., 1997). The existence of a complementary ANP-mediated mechanism to inhibit cell growth and proliferation is not anticipated. Nevertheless, the inhibition of cell proliferation is often accompanied by an increased probability of apoptosis, whereas, growth-promoting agents and agonist hormones tend to promote cell growth and proliferation. For instance, ANG II inhibits apoptosis, in contrast, ANP and nitric oxide, both potently inhibit cell growth and proliferation and induce apoptosis (Pollman et al., 1996; Wu et al., 1997). It has been suggested that the anti-apoptotic molecule Bcl-2 homolog Mcl-1 might serve as an important target in ANP-induced apoptosis. Intriguing was the finding that the Bcl-2 homolog Mcl-1 was initially identified as a protein marker, which was up-regulated during the differentiation of the monocytoid cell line ML-1 cells (Kozopas et al., 1993; Kiefer et al., 1995; Wu et al., 1997).

## GENE-TARGETING OF *Nppa* AND *Npr1*

Genetic-targeting strategies in mice have provided novel approaches to study the physiological responses corresponding to gene-dosage *in vivo* (Takahashi and Smithies, 1999; Kim et al., 2002). Genetically modified mice carrying *Npr1* gene-disruption or gene-duplication have provided strong support for the physiological roles of NPs and their receptors in the intact animals (John et al., 1995; Lopez et al., 1995; Kishimoto et al., 1996; Oliver et al., 1997, 1998; Matsukawa et al., 1999; Pandey et al., 1999; Shi et al., 2001, 2003; Holtwick et al., 2002; Vellaichamy et al., 2005; Das et al., 2012; Zhao et al., 2013). Numerous studies have examined the quantitative contributions and possible mechanisms mediating the responses of *Npr1* gene copies by determining the renal plasma flow (RPF), glomerular filtration rate (GFR), urine flow, and sodium excretion following blood volume expansion in *Npr1* homozygous null mutant (*Npr1*^-/^*^-^;* 0-copy), wild-type (*Npr1*^+/+^*;* 2-copy), and gene-duplicated (*Npr1*^++/++^*;* 4-copy) mice in a *Npr1* gene-dose-dependent manner (Shi et al., 2003). Although, the blood volume expansion stimulated the release of ANP in all three *Npr1* genotypes of mice, significant functional responses (RPF, GFR, and sodium excretion) occurred only in *Npr1^-/-^* and *Npr1*^++/++^ mice but not in *Npr1^-/-^* mice. These findings demonstrated that the ANP/NPRA axis is primarily responsible for mediating the renal hemodynamic and sodium excretory responses to intravascular blood volume expansion. ANP responses to volume expansion led to the significantly lesser excretion of Na^+^ and water in 0-copy null mutant mice and significantly greater excretory responses along with reduced tubular reabsorption in 4-copy mice as compared with 2-copy wild-type mice. Similarly, during the volume expansion, urinary cGMP concentration was significantly lower in null mutant mice and greater in gene-duplicated mice. Our previous findings have established that NPRA is a hallmark receptor, which plays a critical role in mediating the natriuresis, diuresis, and renal hemodynamic responses to acute blood volume expansion (Shi et al., 2003).

Genetic mouse models with disruption of both *Nppa* and *Npr1* genes have provided strong support for the role of this hormone-receptor system in the regulation of blood pressure, cardiac hypertrophy, and other physiological functions (John et al., 1995; Lopez et al., 1995; Oliver et al., 1997, 1998; Melo et al., 1999; Pandey et al., 1999; Shi et al., 2001, 2003; Holtwick et al., 2002; Vellaichamy et al., 2005; Kishimoto et al., 2011; Pandey, 2011). Therefore, the genetic defects that reduce the activity of ANP and its receptor system can be considered as candidate contributors to essential hypertension and CHF (John et al., 1995; Pandey et al., 1999; Zhao et al., 1999; Knowles et al., 2001; Holtwick et al., 2002; Shi et al., 2003; Vellaichamy et al., 2005). Interestingly, complete absence of NPRA causes hypertension in mice and leads to altered renin and ANG II levels, cardiac hypertrophy, and lethal vascular events similar to those seen in untreated human hypertensive patients (Oliver et al., 1997; Shi et al., 2001, 2003; Zhao et al., 2007). In contrast, increased expression of *Npr1* reduces the blood pressures and inflammatory responses, protects heart, and increases the intracellular second messenger cGMP concentrations corresponding to the increasing number of *Npr1* gene copies (Oliver et al., 1998; Pandey et al., 1999; Shi et al., 2003; Vellaichamy et al., 2007, 2014; Zhao et al., 2013). Recent evidence also indicates that CNP and its receptor NPRB can play important role in regulating the cardiac hypertrophy and remodeling as a potential drug target for the treatment of cardiovascular diseases (Del Ry, 2013).

## CONCLUSION

The field of NPs has been advanced to examine the function and signaling mechanisms of their receptors and the role of second messenger cGMP in physiology and pathophysiology of hypertension, renal hemodynamics, cardiovascular functions, and neural plasticity. The development of gene-knockout and gene-duplication mouse models along with transgenic mice have provided a framework for understanding both the physiological and pathophysiological functions of NPs and their receptors in the intact animals *in vivo*. Although, a considerable progress has been made, the transmembrane signal transduction mechanisms of NPs and their receptors remain unresolved. Future investigations should include; the identification and characterization of cellular targets of intracellular second messenger cGMP produced by NPs, including cytosolic and nuclear proteins, role in gene transcription, cell growth and proliferation, apoptosis, and differentiation. A more vigorous studies of the crosstalk with other signaling mechanisms namely, PKC, MAPKs, cAMP, Ca^2+^, and IP_3_ needs to be pursued systematically. NPs are considered as circulating markers of CHF, however, their therapeutic potential for the treatment of cardiovascular diseases such as hypertension, renal insufficiency, cardiac hypertrophy, CHF, and stroke is still lacking. The ultimate goal of the investigations is this field is to fully appreciate the mechanisms of cGMP generation after ligand binding to GC-coupled receptors and the pathways leading to elicit cellular and physiological functions in relation to other signaling molecules with special emphasis to Ca^2+^, IP_3_, and cAMP levels. Identification of the discrete switch points in signal transmission of NPs and their cognate receptors that specify unique directional responses need to be vigorously pursued.

## Conflict of Interest Statement

The author declares that the research was conducted in the absence of any commercial or financial relationships that could be construed as a potential conflict of interest.
